# Impact of Model Parameterisation and Variance Component Estimates on Genomic Predictions of Carcass Traits in Montana Composite Cattle

**DOI:** 10.1111/jbg.70032

**Published:** 2025-11-24

**Authors:** Caroline Assis Almeida, Felipe Eguti de Carvalho, Flávia Cristina Bis, Rachel Santos Bueno Carvalho, Elisângela Chicaroni de Mattos, Rafael Espigolan, Joanir Pereira Eler, Luís Telo da Gama, Fernando Baldi, José Bento Sterman Ferraz

**Affiliations:** ^1^ Faculty of Zootechnics and Food Engineering University of São Paulo Pirassununga São Paulo Brazil; ^2^ Federal University of Santa Maria Palmeira das Missões Rio Grande do Sul Brazil; ^3^ University of Lisbon Lisbon Portugal

**Keywords:** accuracy, crossbred animals, genetic variance, genomic selection

## Abstract

This study evaluated the influence of variance component (VC) estimates, obtained from different models and two relationship matrices, pedigree‐based (BLUP) and genomic information‐based (ssGBLUP), on genomic predictions of carcass traits in Montana composite cattle. Phenotypic records from 14,422 animals were analysed for ribeye area, rump fat thickness, backfat thickness and marbling, along with pedigree information from 193,129 animals and genomic data from 3911 animals genotyped with 49,457 SNPs. Variance components and heritability estimates were calculated using restricted maximum likelihood under single‐trait linear models. Across five models (M1–M5), fixed effects included contemporary group, embryo transfer, age at ultrasound and cow age at calving, while random effects included direct genetic effect and residual. From model M2 onwards, biological type, heterosis and both combined and specific recombination effects were also considered. The Akaike information criterion (AIC) was used to identify the best‐fitting model. Different VC estimates were applied in ssGBLUP predictions to evaluate predictive ability based on accuracy, bias and dispersion. Variance component and heritability estimates were similar between methods, although ssGBLUP yielded higher direct additive genetic variances and heritabilities. More parameterised models using ssGBLUP provided a better fit according to AIC. However, less parameterised models showed superior predictive ability, regardless of whether VCs were estimated by BLUP or ssGBLUP. When comparing predictive ability across sources, pedigree‐based VC estimates resulted in more accurate predictions. Thus, the choice of model complexity should be guided by the analysis objective and the available data structure.

## 1 Introduction

The Montana composite cattle was developed through the crossbreeding of two bovine subspecies, combining distinct characteristics of *Bos indicus* and *Bos taurus* . This crossbreeding aimed to enhance both productivity and adaptation to tropical and subtropical climates (Ferraz et al. 1999), allowing the use of composite bulls in natural matings in those regions, with better fertility and longevity than *Bos taurus* bulls (Oliveira et al. 2024). The genetic composition of this composite population integrates four biological types, which classify breeds into groups according to similarities in physiology, function, reproduction and growth traits: N ( *Bos indicus* origin), A ( *Bos taurus* animals adapted to tropical environments), B ( *Bos taurus* of British origin) and C ( *Bos taurus* of Continental origin) (Ferraz et al. 1999; Oliveira et al. 2024).

By capitalising on the genetic effects of heterosis and complementarity, the Montana composite breed combines desirable traits such as meat quality and sexual precocity from taurine breeds with the heat tolerance and disease resistance of zebu breeds, thereby resulting in hybrid vigour and greater productivity (Grigoletto et al. 2020). Another key concept in composite populations is the recombination of genetic effects, which plays a critical role in increasing genetic variability within the population (Stapley et al. 2017).

To maximise livestock productive potential, it is essential to accurately estimate variance components and genetic parameters, as these metrics underpin the selection of superior animals (Falconer and Mackay 1996). In this context, the inclusion of additional effects in the models, such as biological types and non‐additive effects, including heterosis and recombination, may be critical for reducing the influence of environmental factors and avoiding biased estimates in composite populations (Oliveira et al. 2024). In these sense, ignoring these effects in the model would inflate heritability estimates and bias breeding value predictions, potentially compromising selection decisions and the efficiency of breeding programmes.

In studies applying the animal model to this population, heterosis has often been included as a fixed effect (Grigoletto et al. 2020; Kluska et al. 2021), however, recombination has not been considered. According to Oliveira et al. (2024), both heterosis and recombination should be accounted for in the models, as they can influence the prediction of breeding values. Recombination is a fundamental mechanism for generating genetic variability through crossing‐over (Arnheim et al. 2007). It plays a crucial role in breaking down linkage disequilibrium, thereby increasing genetic variance and improving the response to selection, while ignoring recombination can either limit or inflate the genetic variance, ultimately affecting the accuracy of selection decisions (Barton and Otto 2005). Moreover, its rate and pattern can vary across species, populations and individuals, and are influenced by environmental factors (Stapley et al. 2017) which underscores the importance of including recombination in genetic models, particularly in composite populations.

The use of genomic information has already become a common practice in cattle breeding programmes, reducing the generation interval, increasing the accuracy of genetic parameter estimation, and the prediction of young animals, and consequently accelerating genetic gain (Albuquerque et al. 2017; Magalhães et al. 2019; Neves et al. 2014; Xu et al. 2020; Zhang et al. 2014). However, despite the benefits of genomic prediction and the resulting genetic gains, there is still no consensus on whether genomic information should be used to estimate variance components. This uncertainty can lead to practical consequences, including inconsistent heritability estimates and potential bias in selection decisions. One contributing factor is that variance components estimated using the pedigree‐based relationship matrix and the single‐step genomic relationship matrix are often very similar while the latter requires substantially higher computational resources.

Consequently, selection has relied on pedigree‐based data, while genomic data are primarily applied only after genomic selection (Cardona‐Cifuentes et al. 2024; Jensen 2016). Even when using the single‐step genomic best linear unbiased predictor (ssGBLUP) to estimate variance components, bias may arise due to positive assortative mating (inbreeding) and selective genotyping in the identification and selection of superior candidates, which can rapidly reduce genetic diversity (Gowane et al. 2019; Vanraden 2020; Wang et al. 2020). Estimates based on the traditional pedigree‐based relationship matrix are biased because not all phases of selection are considered (Aldridge et al. 2020; Hidalgo et al. 2020; Wang et al. 2020).

The use of the genomic relationship matrix (**G**) (Vanraden 2008) in variance component estimation may depend on population structure, marker density and the representativeness of the genotypic panel relative to the base population (Misztal et al. 2020; Uemoto et al. 2015; Van Grevenhof and Van der Werf 2015). These shifts in population structure raise important questions about whether variance component estimates obtained from data collected prior to genomic selection are still representative and applicable to current scenarios. Thus, when using **G** for variance component estimation, the results reflect a specific and contemporary population, rather than the classical population parameters defined by traditional quantitative theory (Goddard and Hayes 2009; Yang et al. 2010).

In this context, given the lack of comparative and methodological studies on the use of genomic information for estimating variance components, particularly in breeds such as the Montana composite, this study aims to estimate variance components for carcass traits obtained by ultrasonography using two relationship matrices (pedigree and genomic based) and five genetic models that incorporate biological type effects and non‐additive effects. Furthermore, it evaluates the variance component estimates and their impact on breeding value predictions and seeks to identify the most appropriate model for the traits studied.

## 2 Materials and Methods

### 2.1 Database

The datasets from the Montana Composite breeding programme were provided by the Montana Composite Genetic Improvement Programme and were stored and analysed by the Animal Breeding and Biotechnology Group (GMAB) at the Faculty of Animal Science and Food Engineering (FZEA), University of São Paulo (USP), Brazil.

#### 2.1.1 Phenotypic Database

The phenotypic database included records from 14,422 Montana Composite animals with ultrasound‐based carcass measurements, from individuals born between 2008 and 2022. The traits analysed were rib eye area (REA, cm^2^), rump fat (RF, mm), back fat thickness (BF, mm) and intramuscular fat percentage (IMF, %). REA, BF and IMF were measured on the *Longissimus dorsi* muscle between the 12th and 13th ribs, whereas RF was measured at the rump, between the ilium and ischium, at the *Gluteus medius* and *Biceps femoris* muscle intersections. All traits were evaluated by ultrasonography using an ALOKA 500V device equipped with a 3.5MHz linear probe. The contemporary groups (CG) were composed by year of birth, and the farm and management group where the measurement was recorded. The CGs with fewer than 10 animals were excluded from subsequent analyses. Descriptive statistics of carcass phenotypes after quality control are presented in Table 1.

**TABLE 1:** Descriptive statistics for carcass traits evaluated in animals from Montana.

Traits	*N*	Mean	SD	Min	Max	CV (%)	nCG
REA (cm^2^)	14,404	62.02	14.20	18.60	122.30	22.90	225
RF (mm)	14,044	3.90	2.29	0.30	47.70	58.67	225
BT (mm)	13,452	2.95	1.47	0.30	19.20	49.69	224
IMF (%)	4575	2.88	1.14	0.32	8.59	39.40	88

Abbreviations: BF, back fat thickness; CV, coefficient of variation; IMF, intramuscular fat percentage; Max, maximum value; Min, minimum value; *N*, number of individuals; nCG, number of contemporary groups; REA, rib eye area; RF, rump fat; SD, standard deviation.

#### 2.1.2 Pedigree Database

The pedigree file comprised 193,129 animals, including 3682 bulls and 97,939 dams, with 101,621 individuals having progeny and 91,508 individuals without progeny. Among the bulls, 1016 were founders (sires of 7320 offspring), and among the dams, 18,175 were founders (dams of 18,250 offspring). The average inbreeding coefficient was 0.015, observed in 29,768 animals with some level of inbreeding, and was calculated using CFC programme (Sargolzaei et al. 2006). The pedigree covered an average of 2.49 generations, with a maximum depth of 9.89 generations; however, only 3 generations were fully traced.

The pedigree included all biological types that form the Montana genetic base (N, A, B and C), as well as crossbred individuals among these types. Type N comprises breeds adapted to tropical conditions, notably parasite‐resistant and heat‐tolerant, with a predominance of Nellore, in addition to Brahman, Gir, Guzerá, Sindi and Tabapuã. Type A includes breeds adapted to tropical environments and characterised by good fertility, with significant contributions from Bonsmara and Senepol, along with Belmont Red, Caracu and Tuli. Type B consists of breeds recognised for their sexual precocity, carcass quality and high growth rate, with Red Angus being the most representative, followed by Aberdeen Angus, Devon, Hereford and South Devon. Finally, Type C encompasses breeds characterised by high growth rates and carcass quality, such as Charolais, Gelbvieh, Limousin, Marchigiana and Simmental, with the latter exerting the greatest influence.

#### 2.1.3 Genomic Datasets

The genomic dataset consisted of 3911 genotyped animals (3639 males and 272 females) using the medium‐density SNP panel GeneSeek Genomic Profiler Bovine LD v4 (Illumina Inc., San Diego, CA), which contained 51,686 SNPs. Quality control procedures were performed with the default PREGSF90 program (Aguilar et al. 2014), removing markers with a call rate < 0.90, minor allele frequency (MAF) < 0.05, monomorphic SNPs, and extreme deviation from Hardy–Weinberg equilibrium by the maximum difference between the observed and expected frequency of heterozygosity > 0.15. After quality control, 49,474 SNPs remained for subsequent analyses. The distribution of genotyped animals across birth years is presented in Figure S1.

### 2.2 Estimation of Variance Components and Genetic Parameters

Variance components and genetic parameters were estimated using five linear models that incorporated either pedigree or genomic information. Best Linear Unbiased Prediction (BLUP, Henderson 1975) and ssGBLUP (Aguilar et al. 2010; Christensen and Lund 2010) were used to estimate variance components. Single‐trait analyses were performed using the restricted maximum likelihood (REML) method with the Average Information (AI) algorithm, which employs the second derivative of the likelihood function and provides faster convergence. All analyses were conducted using the BLUPF90+ program from the BLUPF90 program family (Lourenco et al. 2022; Misztal et al. 2014).

In all models, the fixed effects included contemporary group, embryo transfer (meaning whether the individual was produced by embryo transfer or not), animal age at ultrasound (average 573.03 ± 63.49 days) and cow age at calving (linear and quadratic covariates), whereas the direct genetic effect of the animal and the residual were considered random effects. From the second animal model onward, breed composition was incorporated through biological type effects, expressed as percentages: N (0.22 ± 0.10), A (0.50 ± 0.17), B (0.24 ± 0.16) and C (0.09 ± 0.06), in addition to combined direct heterosis (0.66 ± 0.15) and combined direct recombination (0.59 ± 0.12).

Specific direct heterosis and specific direct recombination between pairs of biological types were also included. The mean percentages and standard deviations for specific heterosis and recombination were as follows: N × A (0.19 ± 0.08; 36.12 ± 5.22), N × B (0.10 ± 0.09; 322.57 ± 219.93), N × C (0.04 ± 0.05; 141.41 ± 23.08), A × B (0.20 ± 0.10; 118.36 ± 20.24), A × C (0.08 ± 0.05; 0.17 ± 0.08) and B × C (0.04 ± 0.04; 0.10 ± 0.08). The models evaluated are presented in Table 2.

**TABLE 2:** Description of the models tested and their included effects.

Models	
M1	$y_{\text{ijklmn}}={\mathrm{CG}}_{i}+{\mathrm{ET}}_{j}+\mathrm{Age}\_{\mathrm{us}}_{k}+\beta_{1}\cdot \mathrm{Age}\_{\mathrm{cow}}_{l}+{\beta_{2}\cdot \mathrm{Age}\_\mathrm{cow}}_{m}^{2}+u_{n}+e_{\text{ijklmn}}$,
M2	$y_{\text{ijklmnop}}={\mathrm{CG}}_{i}+{\mathrm{ET}}_{j}+\mathrm{Age}\_{\mathrm{us}}_{k}+\beta_{1}\cdot \mathrm{Age}\_{\mathrm{cow}}_{l}+{\beta_{2}\cdot \mathrm{Age}\_\mathrm{cow}}_{m}^{2}+{\text{NABC}}_{n}+{\mathrm{Het}}_{o}+u_{p}+e_{\text{ijklmnop}}$,
M3	$y_{\text{ijklmnopq}}={\mathrm{CG}}_{i}+{\mathrm{ET}}_{j}+\mathrm{Age}\_{\mathrm{us}}_{k}+\beta_{1}\cdot \mathrm{Age}\_{\mathrm{cow}}_{l}+{\beta_{2}\cdot \mathrm{Age}\_\mathrm{cow}}_{m}^{2}+{\text{NABC}}_{n}+\;{\mathrm{Het}}_{o}+{\text{Recomb}}_{p}+u_{q}+e_{\text{ijklmnopq}}$,
M4	$y_{\text{ijklmnopq}}={\mathrm{CG}}_{i}+{\mathrm{ET}}_{j}+\mathrm{Age}\_{\mathrm{us}}_{k}+\beta_{1}\cdot \mathrm{Age}\_{\mathrm{cow}}_{l}+{\beta_{2}\cdot \mathrm{Age}\_\mathrm{cow}}_{m}^{2}+{\text{NABC}}_{n}+{\text{Recomb}}_{o}+\text{Specific}\_{\mathrm{het}}_{p}+u_{q}+e_{\text{ijklmnopq}}$,
M5	$y_{\text{ijklmnopq}}={\mathrm{CG}}_{i}+{\mathrm{ET}}_{j}+\mathrm{Age}\_{\mathrm{us}}_{k}+\beta_{1}\cdot \mathrm{Age}\_{\mathrm{cow}}_{l}+{\beta_{2}\cdot \mathrm{Age}\_\mathrm{cow}}_{m}^{2}+{\text{NABC}}_{n}+\text{Specific}\_{\mathrm{het}}_{o}+\text{Specific}\_{\text{recomb}}_{p}+u_{q}+e_{\text{ijklmnopq}}$,

*Note:* M1: model 1; M2: model 2; M3: model 3; M4: model 4; M5: model 5; Age_us: age of the animal at ultrasound; Age_cow: age of the cow at calving as a linear effect; Age_cow^2^: age of the cow at calving as a quadratic effect; *β*_1_ and *β*_2_ are the regression coefficients; NABC: proportional contribution of NABC biological types to the genetic composition of the individual; Het: combined direct heterosis; Recomb: combined direct recombination; Specific_het: direct heterosis between biological types; and Specific_recomb: direct recombination between biological types.

Abbreviations: CG, contemporary group; ET, embryo transfer.

It was assumed that heterosis is linearly related to the degree of heterozygosity, calculated as the proportion of loci in an individual that are expected to carry alleles originating from different breeds. The heterozygosity resulting from the specific combination of breeds *i* and *j* in a given individual was calculated according to Dickerson (1969):

(1) $$\mathrm{He}t_{\mathrm{ij}}=\alpha_{i}^{s}\alpha_{j}^{d}+\alpha_{j}^{s}\alpha_{i}^{d},$$

where $\alpha_{i}^{s}$ and $\alpha_{j}^{d}$ represent the proportions contributed by breed *i* to the sire and by breed *j* to the dam of the individual, respectively. A direct combined estimate of heterozygosity was calculated according to Vanraden and Sanders (2003):

(2) $$\mathrm{Het}=1-\sum_{i=1}^{n}\left(\alpha_{i}^{s}\times \alpha_{i}^{d}\right),$$

Therefore, the recombination effect was proposed as a broader concept, representing the breakdown of epistatic combinations observed in the progeny of crossbred parents (Kinghorn 1980). The coefficient for recombination effects resulting from the use of a sire and dam with genetic contributions from breeds *i* and *j* ($\alpha_{i}^{s}$ and $\alpha_{j}^{s}$, respectively) was calculated as:

(3) Recij=αis+αjdαjs+αid−Hetij,

A direct combined estimate of the coefficient of recombination effects resulting from the genetic contributions of multiple breeds was obtained as:

(4) $$\mathrm{Rec}=1-\sum_{i=1}^{n}\frac{{\left(\alpha_{i}^{s}\right)}^{2}+{\left(\alpha_{i}^{d}\right)}^{2}}{2},$$

The model used for all characteristics can be represented as:

(5) y=Xβ+Zu+e,

where y is the vector of phenotypic observations; β represents the vector of fixed effects; u represents the direct additive genetic effects; e represents the vector of residual random effects; X and Z are the incidence matrices for the fixed and direct additive genetic effects, respectively. It is assumed that $E\left[y\right]=\mathrm{X\beta}$, where the direct additive genetic effects and residuals follow a normal distribution with mean zero. For the direct additive genetic effects, it is assumed that $\mathrm{Var}\left(u\right)=A⊗S_{u}$ or $\mathrm{Var}\left(u\right)=H⊗S_{u}$, where A is numerator relationship matrix constructed from pedigree information, H is the pedigree–genomic relationship matrix and $S_{u}$ is the additive genetic covariance matrix. For the residuals, it is assumed that $\mathrm{Var}\left(e\right)=I⊗S_{e}$, where I is an appropriate order identity matrix and $S_{e}$ is the residual covariance matrix.

In ssGBLUP, the inverse of the pedigree‐based relationship matrix $A^{-1}$ is replaced by the inverse of the combined pedigree–genomic matrix $H^{-1}$ (Aguilar et al. 2010; Christensen and Lund 2010). The **H** matrix integrates both pedigree and genomic information, and its inverse is obtained as follows:

(6) $$H^{-1}=A^{-1}+\left[\begin{array}{cc} 0 & 0\\ 0 & {\mathrm{\tau G}}^{-1}+{\mathrm{\omega A}}_{22}^{-1} \end{array}\right],$$

where **H** represents the matrix of relationship coefficients among animals, $A_{22}^{-1}$ is the inverse of the pedigree‐based relationship matrix for genotyped animals, and $G^{-1}$ is the inverse of the genomic relationship matrix, assumed as τ = 1.0 and ω = 1.0, constructed according to VanRaden's method I (Vanraden 2008).

#### 2.2.1 Comparison of Models

To identify the most appropriate model for estimating variance components among the five animal models proposed, with or without the use of genomic information, the Akaike Information Criterion (AIC) was applied, defined as:

(7) $$\mathrm{AIC}=-2\ln\left(L\right)+2p,$$

where $\ln\left(L\right)$ represents the value of the likelihood function, and p represents the number of parameters estimated in the model (Akaike 1973). Model selection followed the guidelines of Burnham and Anderson (2002), which assess the relative loss of information when using a given model *i* compared with the model considered most appropriate, allowing comparisons among both nested and non‐nested models. According to these guidelines, an AIC difference less than or equal to 2 indicates substantial support for competing models, values between 4 and 7 suggest considerably less support, and values greater than 10 imply essentially no support for the model with the higher AIC.

The Bayesian Information Criterion (BIC) was also used for model comparison (Schwarz 1978). BIC is defined as:

(8) $$\mathrm{BIC}=-2\ln\left(L\right)+\ln\left(n\right)\cdot p,$$

where n is the sample size. Both criteria (AIC and BIC) are based on the maximum likelihood estimate and include a penalty term to discourage overparameterisation, however they differ in how this penalty is applied.

In addition, the likelihood ratio test (LRT) was used to evaluate the goodness of fit between two models, where a more parameterised model is compared with a less parameterised one. The purpose of this test is to determine whether the inclusion of additional parameters significantly improves the model fit. The LRT is based on the difference in log‐likelihood between the two models, which follows a chi‐squared distribution with degrees of freedom equal to the difference in the number of estimated parameters (Felsenstein 1981; Huelsenbeck and Crandall 1997). The test statistic can be expressed as follows:

(9) $$\mathrm{LRT}=-2\cdot \left[\ln\left(L_{\text{less parameterised}}\right)-\ln\left(L_{\text{more parameterised}}\right)\right],$$

### 2.3 Prediction Ability

For the forward‐validation analyses, the dataset was divided into two subsets: partial (p) and whole (w). In the partial subset, the genomic prediction (GEBV_p_) was performed based on pedigree and genotype information, without including the phenotypes of the validation animals. In the complete subset, the phenotypes of the validation animals were included, and the full genomic estimated breeding value (GEBV_w_) was predicted using all available information: phenotypes, genotypes and pedigree.

Animals born in 2021 and 2022 were selected as the validation group (p) because they were young selection candidates without progeny but with both phenotypic and genotypic data available. This validation subset included 369, 368, 366 and 147 animals for the REA, RF, BF and IMF traits, respectively. GEBVs were predicted for all traits under a single‐trait model, according to Equation (5), using variance components estimated without genomics (BLUP) and with genomics (ssGBLUP).

Accuracy, bias and dispersion were calculated according to the methodology proposed by Legarra and Reverter (2018). Prediction accuracy was defined as:

(10) $${\hat{\rho }}_{w,p}=\sqrt{\frac{\mathrm{cov}\left({\hat{u}}_{w},{\hat{u}}_{p}\right)}{\left(1-\bar{F}\right)\cdot {\hat{\sigma }}_{u}^{2}},}$$

where $\mathrm{cov}\left({\hat{u}}_{w},{\hat{u}}_{p}\right)$ is the covariance between the vector of predicted breeding values for the validation animals using the whole dataset (${\hat{u}}_{w}$) and the vector of predicted breeding values for the validation animals using the partial dataset (${\hat{u}}_{p}$), $\bar{F}$ is the mean inbreeding coefficient estimated with the CFC program (Sargolzaei et al. 2006) and ${\hat{\sigma }}_{u}^{2}$ is the direct additive genetic variance for each trait studied.

Dispersion was assessed by regressing the GEBVs from the whole dataset on those from the partial dataset. In the absence of inflation or deflation of the predicted breeding values, the expected regression coefficient is equal to 1.

(11) $${\hat{b}}_{w,p}=\frac{\mathrm{cov}\left({\hat{u}}_{w},{\hat{u}}_{p}\right)\;}{\mathrm{var}\left({\hat{u}}_{p}\right)},$$

Bias was calculated as the difference between the partial and whole GEBVs, standardised by the direct additive genetic standard deviation of each trait. The expected value of this estimator is zero when no bias is present in the evaluation:

(12) $${\hat{\mu }}_{w,p}=\frac{{\hat{u}}_{p}-{\hat{u}}_{w}\;}{\sqrt{{\hat{\sigma }}_{u}^{2}}},$$

## 3 Results

### 3.1 Estimation of Variance Components and Heritability Coefficients

The estimates of variance components were similar across models and methods (BLUP and ssGBLUP) for all analysed traits (Table 3). The highest direct additive genetic variance was consistently associated with the ssGBLUP method. This difference was most pronounced for REA, with increases of 0.770 (M1), 0.995 (M2), 0.953 (M3), 0.916 (M4) and 0.885 (M5) in the additive variance estimates obtained with ssGBLUP compared to BLUP. Heritability estimates ranged from 0.203 (M2_BLUP_) to 0.230 (M5_ssGBLUP_) for REA, 0.173 (M4_BLUP_) to 0.205 (M1_ssGBLUP_) for RF, 0.127 (M5_BLUP_) to 0.162 (M1_ssGBLUP_) for BF and 0.390 (M4_BLUP_) to 0.449 (M1_ssGBLUP_) for IMF.

**TABLE 3:** ‐ Estimates of variance components and heritability coefficients for rib eye area (REA), rump fat thickness (RF), back fat thickness (BF) and intramuscular fat percentage (IMF), obtained using BLUP and ssGBLUP across different models (mean ± standard error) in Montana composite cattle.

Trait	Model	${\hat{\sigma }}_{u}^{2}$	${\hat{\sigma }}_{e}^{2}$	${\hat{h}}^{2}$	AIC	BIC
REA	M1_BLUP_	13.151 ± 1.358	48.142 ± 1.190	0.215 ± 0.021	98,898.407	98,913.558
	M1_ssGBLUP_	13.921 ± 1.267	47.368 ± 1.097	0.227 ± 0.0195	98,798.814	98,813.965
	M2_BLUP_	12.362 ± 1.323	48.602 ± 1.174	0.203 ± 0.021	98,841.487	98,856.638
	M2_ssGBLUP_	13.357 ± 1.249	47.655 ± 1.090	0.219 ± 0.019	98,745.457	98,760.608
	M3_BLUP_	12.469 ± 1.328	48.507 ± 1.176	0.205 ± 0.021	98,836.858	98,852.009
	M3_ssGBLUP_	13.422 ± 1.251	47.590 ± 1.090	0.220 ± 0.019	98,739.561	98,754.712
	M4_BLUP_	12.704 ± 1.342	48.332 ± 1.182	0.208 ± 0.021	98,813.800	98,828.951
	M4_ssGBLUP_	13.620 ± 1.259	47.456 ± 1.094	0.223 ± 0.020	98,718.667	98,733.818
	M5_BLUP_	12,992 ± 1.350	47.417 ± 1.180	0.215 ± 0.021	98,669.460	98,684.611
	M5_ssGBLUP_	13.877 ± 1.262	46.558 ± 1.087	0.230 ± 0.020	98,569.626	98,584.777
RF	M1_BLUP_	0.425 ± 0.049	1.937 ± 0.045	0.180 ± 0.020	51,523.583	51,538.683
	M1_ssGBLUP_	0.485 ± 0.049	1.885 ± 0.043	0.205 ± 0.020	51,444.750	51,459.850
	M2_BLUP_	0.413 ± 0.048	1.938 ± 0.044	0.176 ± 0.020	51,473.418	51,488.518
	M2_ssGBLUP_	0.476 ± 0.049	1.886 ± 0.043	0.202 ± 0.020	51,405.010	51,420.110
	M3_BLUP_	0.413 ± 0.048	1.937 ± 0.044	0.176 ± 0.020	51,465.034	51,480.134
	M3_ssGBLUP_	0.477 ± 0.049	1.883 ± 0.043	0.202 ± 0.020	51,395.910	51,411.010
	M4_BLUP_	0.407 ± 0.048	1.942 ± 0.044	0.173 ± 0.020	51,459.202	51,474.302
	M4_ssGBLUP_	0.471 ± 0.048	1.888 ± 0.043	0.200 ± 0.020	51,390.838	51,405.938
	M5_BLUP_	0.409 ± 0.048	1.934 ± 0.044	0.175 ± 0.020	51,461.168	51,476.268
	M5_ssGBLUP_	0.472 ± 0.049	1.881 ± 0.043	0.201 ± 0.020	51,390.698	51,405.798
BF	M1_BLUP_	0.135 ± 0.018	0.877 ± 0.018	0.134 ± 0.017	38,257.339	38,272.353
	M1_ssGBLUP_	0.164 ± 0.019	0.852 ± 0.018	0.162 ± 0.018	38,195.730	38,210.744
	M2_BLUP_	0.132 ± 0.018	0.877 ± 0.018	0.130 ± 0.017	38,217.343	38,232.357
	M2_ssGBLUP_	0.160 ± 0.019	0.853 ± 0.018	0.158 ± 0.018	38,167.894	38,182.908
	M3_BLUP_	0.132 ± 0.018	0.876 ± 0.018	0.131 ± 0.017	38,214.804	38,229.818
	M3_ssGBLUP_	0.161 ± 0.019	0.852 ± 0.018	0.159 ± 0.018	38,163.681	38,178.695
	M4_BLUP_	0.129 ± 0.017	0.878 ± 0.018	0.128 ± 0.017	38,206.431	38,221.445
	M4_ssGBLUP_	0.159 ± 0.019	0.853 ± 0.018	0.157 ± 0.018	38,155.618	38,170.632
	M5_BLUP_	0.127 ± 0.017	0.877 ± 0.018	0.127 ± 0.017	38,210.479	38,225.493
	M5_ssGBLUP_	0.156 ± 0.018	0.853 ± 0.018	0.155 ± 0.018	38,160.820	38,175.834
IMF	M1_BLUP_	0.237 ± 0.030	0.329 ± 0.024	0.419 ± 0.048	10,189.918	10,202.775
	M1_ssGBLUP_	0.256 ± 0.029	0.315 ± 0.022	0.449 ± 0.045	10,156.601	10,169.458
	M2_BLUP_	0.219 ± 0.029	0.340 ± 0.024	0.392 ± 0.048	10,159.726	10,172.583
	M2_ssGBLUP_	0.240 ± 0.029	0.324 ± 0.022	0.426 ± 0.045	10,132.461	10,145.318
	M3_BLUP_	0.220 ± 0.030	0.339 ± 0.024	0.393 ± 0.048	10,161.284	10,174.141
	M3_ssGBLUP_	0.241 ± 0.029	0.323 ± 0.022	0.427 ± 0.045	10,133.406	10,146.263

	M4_BLUP_	0.218 ± 0.030	0.341 ± 0.024	0.390 ± 0.048	10,158.353	10,171.210
	M4_ssGBLUP_	0.239 ± 0.029	0.326 ± 0.022	0.423 ± 0.045	10,131.517	10,144.374
	M5_BLUP_	0.219 ± 0.030	0.337 ± 0.024	0.394 ± 0.049	10,164.250	10,177.107
M5_ssGBLUP_	0.239 ± 0.029	0.323 ± 0.022	0.426 ± 0.046	10,138.916	10,151.773

*Note:* ${\hat{\sigma }}_{u}^{2}$: direct additive genetic variance; ${\hat{\sigma }}_{e}^{2}$: residual variance; ${\hat{h}}^{2}$: direct additive genetic heritability coefficient; M1_BLUP_: model 1 without inclusion of genomic information; M1_ssGBLUP_: model 1 with inclusion of genomic information; M2_BLUP_: model 2 without inclusion of genomic information; M2_ssGBLUP_: model 2 with inclusion of genomic information; M3_BLUP_: model 3 without inclusion of genomic information; M3_ssGBLUP_: model 3 with inclusion of genomic information; M4_BLUP_: model 4 without genomic information; M4_ssGBLUP_: model 4 with genomic information; M5_BLUP_: model 5 without genomic information; M5_ssGBLUP_: model 5 with genomic information.

Abbreviations: AIC: Akaike Information Criterion; BIC: Bayesian Information Criterion.

Based on the AIC results, for REA, the M5_BLUP_ and M5_ssGBLUP_ models showed the best fit to the data. For RF, the M5_BLUP_ or M4_BLUP_ models, as well as the M5_ssGBLUP_ or M4_ssGBLUP_ models, were the most appropriate. For BF, the best performance was observed with the M4_BLUP_ and M4_ssGBLUP_ models. For IMF, the M4_BLUP_ or M2_BLUP_ and the M4_ssGBLUP_, M3_ssGBLUP_, or M2_ssGBLUP_ models stood out, indicating less information loss and, therefore, better fit to the observed data.

For all traits, the best AIC values were obtained using the ssGBLUP method and the more parameterised models. When comparing the two estimation methods, the more parameterised ssGBLUP model (M5) showed the lowest AIC values for REA and RF, while the M4 model yielded the lowest values for BF and IMF. Thus, models incorporating genomic information demonstrated a better fit, according to the AIC criterion, for all evaluated traits.

The results for the best fitting models according to BIC were consistent with those obtained using AIC (Table 3). Also, LRT confirmed the statistical significance of the differences between models (Table 4). For REA, both M5_BLUP_ and M5_ssGBLUP_ were identified as the best models, showing significant differences from other models, indicating that the inclusion of effects improves model fit. For fat thickness traits, M5_BLUP_ or M4_BLUP_, as well as the corresponding ssGBLUP models, were the most appropriate models. For IMF, differences between models were smaller and mostly non‐significant, suggesting a limited effect of additional model complexity for this trait.

**TABLE 4:** Likelihood ratio test statistics for rib eye area (REA), rump fat thickness (RF), back fat thickness (BF) and intramuscular fat percentage (IMF) in Montana composite cattle.

Comparisons between models	Trait			
	REA	RF	BF	IMF
M1_BLUP_ vs. M5_BLUP_	228.95*	62.41*	46.86*	25.67
M2_BLUP_ vs. M5_BLUP_	172.04*	12.25	6.87	−4.52
M3_BLUP_ vs. M5_BLUP_	167.40*	3.87	4.33	−2.97
M4_BLUP_ vs. M5_BLUP_	144.34*	3.87	4.05	−5.90
M1_ssGBLUP_ vs. M5_ssGBLUP_	229.19*	54.05*	34.91*	34.91
M2_ssGBLUP_ vs. M5_ssGBLUP_	175.83*	14.31	7.07	7.07
M3_ssGBLUP_ vs. M5_ssGBLUP_	169.94*	5.21	2.86	2.86
M4_ssGBLUP_ vs. M5_ssGBLUP_	149.04*	0.14	−5.20	−5.20

*Note:* M1_BLUP_: model 1 without inclusion of genomic information; M1_ssGBLUP_: model 1 with inclusion of genomic information; M2_BLUP_: model 2 without inclusion of genomic information; M2_ssGBLUP_: model 2 with inclusion of genomic information; M3_BLUP_: model 3 without inclusion of genomic information; M3_ssGBLUP_: model 3 with inclusion of genomic information; M4_BLUP_: model 4 without genomic information; M4_ssGBLUP_: model 4 with genomic information; M5_BLUP_: model 5 without genomic information; M5_ssGBLUP_: model 5 with genomic information.

* *p* < 0.05.

### 3.2 Prediction Ability

The results of predictive ability for all models using the LR method are presented in Table 5. Model M1 generally showed the best performance for the evaluated traits, both when using variance components estimated by BLUP and by ssGBLUP. In other words, the less parameterised models yielded better results in terms of predictive ability.

**TABLE 5:** Accuracy, dispersion and bias of genomic value predictions for rib eye area (REA), rump fat thickness (RF), back fat thickness (BF) and intramuscular fat percentage (IMF) in Montana Composite cattle, using variance components estimated by the BLUP and ssGBLUP methods across five different models.

Trait	Source of variance components	Estimates		
		${\hat{\rho }}_{w,p}$	${\hat{b}}_{w,p}$	${\hat{\mu }}_{w,p}$
REA	M1_BLUP_	0.739	1.018	0.031
	M1_ssGBLUP_	0.732	1.016	0.031
	M2_BLUP_	0.714	1.015	0.032
	M2_ssGBLUP_	0.704	1.012	0.033
	M3_BLUP_	0.721	1.014	0.032
	M3_ssGBLUP_	0.712	1.011	0.033
	M4_BLUP_	0.721	1.007	0.031
	M4_ssGBLUP_	0.713	1.004	0.032
	M5_BLUP_	0.719	1.005	0.029
	M5_ssGBLUP_	0.711	1.002	0.030
RF	M1_BLUP_	0.578	0.926	−0.102
	M1_ssGBLUP_	0.560	0.917	−0.107
	M2_BLUP_	0.557	0.921	−0.104
	M2_ssGBLUP_	0.539	0.912	−0.110
	M3_BLUP_	0.549	0.918	−0.104
	M3_ssGBLUP_	0.531	0.908	−0.109
	M4_BLUP_	0.535	0.910	−0.106
	M4_ssGBLUP_	0.518	0.899	−0.111
	M5_BLUP_	0.545	0.912	−0.106
	M5_ssGBLUP_	0.528	0.902	−0.112
BF	M1_BLUP_	0.533	0.940	−0.034
	M1_ssGBLUP_	0.496	0.986	−0.023
	M2_BLUP_	0.495	0.939	−0.038
	M2_ssGBLUP_	0.478	0.924	−0.042
	M3_BLUP_	0.491	0.940	−0.037
	M3_ssGBLUP_	0.474	0.924	−0.041
	M4_BLUP_	0.482	0.940	−0.039
	M4_ssGBLUP_	0.467	0.924	−0.044
	M5_BLUP_	0.489	0.932	−0.039
	M5_ssGBLUP_	0.473	0.916	−0.044
IMF	M1_BLUP_	0.371	0.948	0.019
	M1_ssGBLUP_	0.368	0.935	0.018
	M2_BLUP_	0.354	0.969	0.014
	M2_ssGBLUP_	0.352	0.952	0.014

	M3_BLUP_	0.355	0.963	0.012
	M3_ssGBLUP_	0.353	0.947	0.012
	M4_BLUP_	0.356	0.982	0.017
	M4_ssGBLUP_	0.354	0.965	0.017
	M5_BLUP_	0.356	0.975	0.014
M5_ssGBLUP_	0.354	0.960	0.013

*Note:* M1_BLUP_: model 1 without inclusion of genomic information; M1_ssGBLUP_: model 1 with inclusion of genomic information; M2_BLUP_: model 2 without inclusion of genomic information; M2_ssGBLUP_: model 2 with inclusion of genomic information; M3_BLUP_: model 3 without inclusion of genomic information; M3_ssGBLUP_: model 3 with inclusion of genomic information; M4_BLUP_: model 4 without inclusion of genomic information; M4_ssGBLUP_: model 4 with inclusion of genomic information; M5_BLUP_: model 5 without inclusion of genomic information; M5_ssGBLUP_: model 5 with inclusion of genomic information; ${\hat{\rho }}_{w,p}$: prediction accuracy; ${\hat{b}}_{w,p}$: dispersion; and ${\hat{\mu }}_{w,p}$: bias.

For all traits, M1 was superior in prediction accuracy (${\hat{\rho }}_{w,p}$), regardless of the source of variance components. However, when directly comparing the information sources, components estimated via BLUP showed better results. Regarding dispersion (${\hat{b}}_{w,p}$), values closest to 1 were observed in models M1 (for RF and BF), M4 (for IMF) and M5 (for REA), mainly with components estimated via BLUP. For BF, model M1_ssGBLUP_ presented the best dispersion value (0.986), differing from the other models, which generally presented values below 0.940, as well as from the pattern observed for the source of the components.

Regarding bias (${\hat{\mu }}_{w,p}$), the best values, those closest to zero, were observed in model M3 for IMF, under both estimation methods (0.012). For the other traits, the less biased predictions were observed in M1 (for RF and BF) and in M5 (for REA). Notably, similar to what was observed for dispersion in BF, the bias in M1_ssGBLUP_ performed better than in BLUP, which was an exception compared with the other models and traits.

## 4 Discussion

### 4.1 Variance Components

The results of this study showed small variations among the models and methods evaluated in the estimation of variance components for carcass traits in Montana Composite cattle, with all heritability estimates remaining within the standard error range. Grigoletto et al. (2020), when evaluating Montana Composite cattle, reported heritability estimates of 0.29 ± 0.03 for REA, 0.26 ± 0.03 for BF and 0.16 ± 0.03 for RF. In the present study, the estimated heritabilities for REA and BF were lower, whereas for RF the values were similar to those reported by these authors. These discrepancies can be attributed mainly to differences in the statistical models adopted: in this study, multiple fixed effects and non‐additive sources of genetic variation were considered, whereas Grigoletto et al. (2020) used a single‐trait model with genomic information, including contemporary group, combined direct heterosis, and animal age at measurement as fixed effects, and direct additive genetic and residual effects as random factors. The complexity and degree of parameterisation of the models adopted directly influence heritability estimates, such that the inclusion of more sources of variation may result in more conservative values by better capturing the variability present in the data since the phenotype is adjusted for different fixed effects, which alters the partitioning of variance components.

The heritability estimates for REA found in this study were lower than those reported for other breeds, with estimates ranging from 0.27 ± 0.00 to 0.52 ± 0.09 (Arikawa et al. 2025; Duff et al. 2021; Novo et al. 2021; Pereira et al. 2023; Pires et al. 2017; Su et al. 2017). Similar estimates to those obtained here were reported by Miar et al. (2014) in crossbred steers (0.17 ± 0.09) and by Ligori et al. (2025) in the Caracu breed (0.24 ± 0.05). For RF, the heritability estimates in this study were like those observed by Ligori et al. (2025), who reported a value of 0.15 ± 0.05. For BF, the estimates were also close to those reported by Pereira et al. (2023), Arikawa et al. (2025) and Ligori et al. (2025), ranging from 0.10 ± 0.00 to 0.15 ± 0.03. For IMF, the heritability estimates obtained in this study were comparable to those reported for breeds such as Hereford, Simmental × Angus crosses, Senepol and Angus, with values ranging from 0.37 ± 0.08 to 0.45 ± 0.04 (Duff et al. 2021; Novo et al. 2021; Su et al. 2017).

In addition to these comparisons, the superiority of more parameterised models in estimating variance components can be attributed to their greater ability to capture additional variation associated with the breed composition of animals, as well as relevant non‐additive effects in composite populations. This result is consistent with expectations for populations such as the Montana Composite, where a history of crossbreeding among distinct biological types can significantly influence both additive and residual genetic components. These observations are supported by Demeke et al. (2003), who demonstrated that, compared with the complete model, ignoring the effects of heterosis and recombination leads to inflation of both direct and maternal genetic variances, as well as inflated direct heritability estimates for all traits in composite populations.

When comparing models with and without genomics for variance component estimation, it can be concluded that models incorporating genomic information provided a better fit, regardless of the trait analysed, as indicated by lower AIC values. For BF and IMF, the differences between the ssGBLUP and BLUP models tended to decrease as the models became more parameterised, suggesting that additional fixed effects partially accounted for variation previously attributed to genomic information. Overall, the inclusion of genomic information improved model fit, particularly in the less parameterised models. This is evident when comparing the AIC of M1_ssGBLUP_ with the BLUP models. For RF, BF and IMF, M1_ssGBLUP_ showed a better fit than M5_BLUP_ and the other models without genomics, while for REA, M1_ssGBLUP_ also showed a better fit than M4_BLUP_ and the less parameterised models. These findings are reinforced by the BIC results and the LRT, which confirmed that the differences between models were statistically significant (*p* < 0.05), further supporting the importance of including relevant effects for improved model fit in REA, RF and BT.

In addition to providing a better fit, the heritability estimated with the ssGBLUP method were higher than those obtained with BLUP. For all traits, the use of the genomic matrix also resulted in higher estimates of direct additive genetic variance and lower estimates of residual variance, as observed by Jang et al. (2022) when comparing the ssGREML (single‐step genomic REML) method with REML. For REA, this approach also reduced standard errors, as reported by Gordo et al. (2016) in the Nellore breed. Therefore, incorporating genomic information into the pedigree‐based relationship matrix may have contributed to correcting the **A** matrix, enabling greater capture of direct additive genetic variance and Mendelian segregation, thereby directly improving model fit.

In general, the inclusion of genomic information in the estimation of variance components can be influenced by several factors, such as genotyping strategy, selection pressure and the proportion of genotyped animals (Jensen 2016; Wang et al. 2020). In the present study, the carcass traits analysed are considered post‐selection, which may introduce bias, as the genotyped animals were predominantly those with superior performance, selected based on their estimated genetic merit. This factor may explain the higher heritability estimates obtained with the ssGBLUP method compared with BLUP (Table 3). In our study, the low proportion of genotyped animals, approximately 2% for each trait, resulted in genomic estimates being only slightly higher or similar those obtained without genomic information, as also reported by Wang et al. (2020).

Another aspect to consider is that the ssGBLUP method relies on the **G** matrix, which is constructed based on observed allele frequencies. However, in composite populations, allele frequency variation may occur among different biological types. According to Makgahlela et al. (2013), the use of breed‐specific allele frequencies in constructing the **G** matrix may compromise the accuracy of genomic relationship estimates, primarily due to the different origins of these frequencies across breeds. Biased relationships may arise from errors in allele frequency estimation, which can also result in biased predicted genomic values, particularly in young animals (Aguilar et al. 2010). Failure to account for these differences may lead to the construction of a **G** matrix that is incompatible with the **A** matrix, thereby compromising the scale of the relationship coefficients and influencing the estimation of variance components, potentially leading to their overestimation (Forni et al. 2011).

### 4.2 Prediction Ability

The less parameterised models showed better predictive ability, as also observed by Scutari et al. (2013), who, when comparing different genomic prediction methods, demonstrated that more parsimonious approaches, such as Elastic Net (EN) combined with relationship matrices, can be as effective as more sophisticated strategies. These findings suggest that, in genomic selection for meat quality, increasing model complexity does not necessarily translate into substantial predictive gains. Thus, it is essential to balance accuracy and parsimony when choosing the most appropriate model.

Based on the results obtained with the M1 models, using BLUP and ssGBLUP components, it appears that the inclusion of multiple effects can reduce information relevant for prediction, particularly in validation subsets with low representation of certain effect levels, likely due to overfitting. This reinforces that less parameterised models tend to be more robust for the selection of young individuals with limited data. This finding aligns with the hypothesis discussed by Legarra and Reverter (2018), that parsimonious models tend to be more robust for genomic evaluation purposes, as they reduce complexity and minimise the risk of overfitting in scenarios with unbalanced or incomplete data.

Although models with genomic information produced higher estimates of direct additive genetic variance and better fit, these gains did not necessarily translate into greater predictive ability, as shown in Table 5. The use of variance components estimated via ssGBLUP may result in more biased predictions, likely due to the additional variance introduced by incorporating genomic information, particularly under selective genotyping and with the low proportion of genotyped animals relative to phenotyped animals.

Similarly, Wang et al. (2020) reported that GEBV inflation may reflect this bias in the estimation of variance components. In their evaluation of genomic predictions using variance components estimated with the **A** and **H** matrices in both real and simulated datasets, the authors observed greater accuracy of GEBVs when components were estimated with the **A** matrix compared with the **H** matrix. These results corroborate the findings of the present study and can be attributed to the composition of the genotyping panel, selective genotyping and bias in the estimation of genetic components under these conditions, factors that affect relationship structure and additive variance estimation, thereby distancing predicted breeding values from the true values and ultimately compromising predictive accuracy.

Gao et al. (2019) also demonstrated that single‐step models, such as ssGBLUP and ssBR (single‐step Bayesian regression), do not accurately estimate variance components in simulated populations undergoing genomic selection. They further reported that pedigree‐based models, particularly when using conventional phase phenotypes or when combined with genomic phase phenotypes, can provide unbiased estimates of these components. Thus, this reinforces the importance of considering not only model complexity but also the method used to estimate variance components, as gains in genomic estimates do not necessarily translate into improved predictive ability.

Finally, the less parametrised models outperformed in prediction ability due to the ability of the relationship matrix to capture effects that are not explicitly modelled but are intrinsic to the animal's genetic value, thus contributing to accurate predictions (Rodríguez‐Almeida et al. 1997). Additionally, less parameterised models are advantageous as they reduce computational demands and shorten analysis time (Bohmanova et al. 2005).

## 5 Conclusion

This study demonstrated that the choice of genetic model and relationship matrix influenced the estimates of variance components and genomic predictions for carcass related traits in Montana Composite cattle. More parameterised models showed a better fit according to AIC for estimating variance components, whereas simpler models yielded greater predictive ability, with higher accuracy, lower bias and better dispersion. The inclusion of genomic information increased the estimates of direct additive genetic variance and heritability but did not necessarily translate into greater predictive ability. For REA and IMF, the impact of genomics was smaller, whereas for RF and BF, model parameterisation had a greater influence. Therefore, the decision regarding the level of complexity to be adopted in the genetic model should be guided by the specific objective of the analysis as well as the structure of the dataset.

## Author Contributions

Caroline Assis Almeida: conceptualisation, formal analysis, writing – original draft, writing – review and editing and visualisation. Felipe Eguti de Carvalho: conceptualisation, writing – original draft and writing – review and editing. Flávia Bis: methodology and writing – review and editing. Rachel Santos Bueno Carvalho: writing – review and editing and supervision. Elisângela Chicaroni de Mattos: data curation, software and resources. Rafael Espigolan and Joanir Pereira Eler: data curation and resources. Luís Telo Da Gama: conceptualisation and writing – review and editing. Fernando Baldi: conceptualisation, validation, supervision, writing – review and editing and project administration. José Bento Sterman Ferraz: conceptualisation, resources, writing – review and editing, supervision and project administration.

## Funding

This study was financed in part by the Coordenação de Aperfeiçoamento de Pessoal de Nível Superior—Brasil (CAPES) ‐ Finance Code 001.

## Ethics Statement

This study was exempt from evaluation by the Animal Ethics Committee (CEUA), as established by Law No. 11794 of 08/10/2008 and Normative Resolution No. 51 of 05/19/2021 from the National Council for Animal Experimentation Control (CONCEA) because the data was obtained from an existing database.

## Conflicts of Interest

The authors declare no conflicts of interest.

## Supporting information


**Figure S1:** Distribution of genotyped animals by year of birth.

## Data Availability

Research data are not shared.
