# Supplementary figures and images for: Impact of Model Parameterisation and Variance Component Estimates on Genomic Predictions of Carcass Traits in Montana Composite Cattle
Source: J Anim Breed Genet. 2025 Nov 24;143(3):431–42. doi: 10.1111/jbg.70032 (PMC13054125; doi:10.1111/jbg.70032)

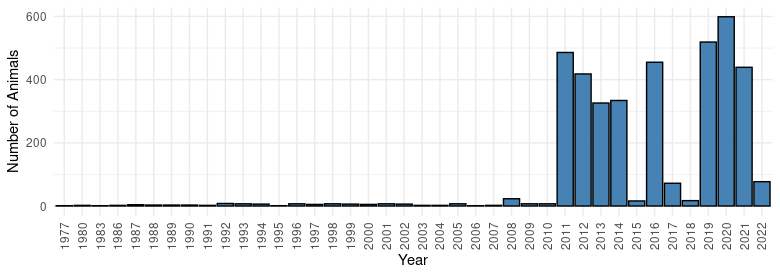


**Figure S1.** Distribution of genotyped animals by year of birth.

Supplement: Supplementary file 1 — Figure S1: Distribution of genotyped animals by year of birth. [file JBG-143-431-s001.docx]
